# The Digital Mental Health Study assessed feasibility of large-scale digital sensing for depression and anxiety

**DOI:** 10.1038/s44482-026-00029-3

**Published:** 2026-08-04

**Authors:** Christopher S. Douglas, Eliza Congdon, Crane Huang, Darsol Seok, Zachary D. Cohen, Samir Akre, Veronica Tozzo, Feiyang Huang, Danielle Ramo-Larios, Raphe A. Bernier, Jonathan Flint, Arash Naeim, Brunilda Balliu, Alex A. T. Bui, Marian Stewart Bartlett, Michelle G. Craske, Nelson B. Freimer

**Affiliations:** 1 University of California, Los Angeles, Depression Grand Challenge, Los Angeles, CA USA; 2 Now at Washington University in St. Louis, St. Louis, MO USA; 3 Apple Inc., Cupertino, CA USA; 4 Now at University of Arizona, Tucson, AZ USA; 5 Now at University of Washington, Seattle, WA USA; 6 University of California, Los Angeles, Center for AI & SMART Health, Los Angeles, CA USA

**Keywords:** Health care, Psychology, Psychology

## Abstract

Data passively obtained from smartphones and wearables can provide nearly continuous objective information that enables quantification of states and traits across broad physiological, behavioral, and emotional domains impacted in mental health conditions, including depression and anxiety. Widespread application of such digital phenotyping could transform the assessment of depression and anxiety in research and clinical care, but the field has lacked well-powered longitudinal studies demonstrating the utility of this approach. This paper describes the design and implementation of the Digital Mental Health Study (DMHS), which collected up to 12 months of sensor data from iPhone and Apple Watch in over 4000 consenting participants, a sample diverse by age, sex at birth, ethnicity, and depression symptom severity. To enable the use of these digital phenotypes to assay the complexity and heterogeneity of depression and anxiety, we designed a protocol of periodic self-report and interview-based scales optimized to assess elements of depression, anxiety, and perceived stress as broadly as possible while minimizing participant measurement burden. We report here the strategies used to recruit and enroll the DMHS sample, the process employed to develop study methods and protocols, and initial findings describing longitudinal symptom trajectories and demonstrating high participant engagement over 12 months.

## Introduction

Depression and anxiety are the most common mental health conditions^1^. They are among the leading contributors to the global burden of disease, affecting several hundred million people worldwide^2^. Reducing this burden will require advances in understanding their underlying causes, developing more effective treatments, and more accurately predicting individual patient outcomes^3–6^.

Current classification frameworks for depression and anxiety largely ignore their heterogeneity, grouping individuals experiencing symptoms into discrete diagnostic categories. For example, a diagnosis of Major Depressive Disorder (MDD) represents more than 200 different possible combinations of symptoms^7,8^. Assessment of depression and anxiety is most commonly based on elicitation of snapshots of symptoms through clinical interviews or self-report scales. These evaluations are subjective, susceptible to inaccurate recall and other biases, and inadequately capture core elements of these conditions (e.g., those relating to physiology or behavior) or their dynamic nature^9^.

The ubiquity of smartphones and wearable devices provides an opportunity to transform the classification and assessment of depression and anxiety, through the continuous collection of multimodal data from their sensors to measure features of physiology, behavior, and emotional states that are associated with these conditions^10^. Such digital phenotyping offers the potential to objectively characterize consenting individuals, on an immense scale. However, despite this potential, the utility of digital phenotyping as a tool for mental health research and clinical care has not yet been convincingly demonstrated^11^.

A series of groundbreaking studies (reviewed by de Angel et al. ^12^) used smartphones and wearables to analyze correlations between depression and anxiety symptoms and digital markers (such as levels of activity, sleep disruption, and social communication patterns). These studies, however, were limited by the following factors: (1) They have focused on small, demographically homogeneous samples and collected data over too short a duration for longitudinal analyses^12,13^. (2) They have not attempted to integrate data from smartphones with those obtainable from wearables, or have analyzed only a fraction of the data streams available on modern devices^14,15^. (3) They have been characterized by inconsistent participant engagement with and adherence to longitudinal protocols in digital mental health research, a factor that has complicated the interpretation of their results^16,17^.

A few recent studies have begun to surmount the above limitations. Notably, RADAR-MDD^18^ (~600 participants with recurrent MDD for up to 2 years) and LifeSense^19^ (~1000 participants with and without MDD for 16 weeks) have demonstrated the feasibility of long-term remote monitoring in clinical cohorts with depression, while AURORA^20^ (~2500 participants experiencing traumatic events) has deployed multimodal sensing and deep phenotyping to monitor neuropsychiatric sequelae specifically following trauma exposure. On an even larger scale, Winbush et al. evaluated the impact of smartphone usage parameters on mental wellbeing indices over a four-week period in over 10,000 individuals^21^. However, to our knowledge no study to date has been of sufficient size or duration to be well-powered to assess the longitudinal relationship between multi-modal digital sensing and depression and anxiety, across the full range of dimensions that are essential elements of these conditions.

To address this knowledge gap, we designed and implemented the Digital Mental Health Study (DMHS), a prospective investigation to identify the relationship between self-reported depression and anxiety symptoms and digital phenotypes obtained from iPhone and Apple Watch. The broader goal of the study was to advance the understanding of causes and trajectories of depression and anxiety through exploration and development of objective measures relevant to these conditions. Prior to conducting the main DMHS study we conducted two small pilot studies to assess feasibility both in terms of participant burden and operational workflows.

In the main DMHS study, we recruited more than 4000 participants who provided up to 12 months of nearly continuous digital data. The sample was diverse by ethnicity, age, sex at birth, and symptom severity. We conducted digital phenotyping by integrating data streams from iPhone with those from Apple Watch, thus obtaining information on a much broader set of domains across physiology, behavior, and emotional state than would be available through other consumer-grade platforms. To determine the utility of these digital phenotypes for assaying the complexity and heterogeneity of depression and anxiety, we designed a protocol of periodic self-report and interview-based scales. The protocol was optimized to assess elements of depression and anxiety as broadly as possible without being too burdensome for participants. Additionally, because both perceived stress and the experience of stressful events are theorized to be risk factors for both depression and anxiety^22^, the DMHS incorporated scales measuring these variables, with the aim of analyzing their relation to both symptom assessments and digital phenotypes.

This paper describes the foundational work of the DMHS, including the process employed to develop the study methods and protocol, recruitment strategies used to enroll the sample, and initial findings demonstrating high participant engagement and adherence over a 12-month period. This information establishes the feasibility of our digital phenotyping approach for conducting large-scale mental health research studies and identifies the elements of depression and anxiety that we aim to illuminate in future analyses of the DMHS data. We suggest how these analyses may advance the development of a novel data-driven framework for unraveling the complexity of these conditions, and of mental health disorders more broadly.

## Results

### Recruitment and enrollment of participants

Between January 2022 and January 2023, 17,150 individuals were screened, resulting in 5684 eligible individuals consenting to participate in the study. From this group, 4542 completed the initial intake assessment (Supplementary Fig. 1), of whom 3333 (73.4% of those completing the initial assessment) maintained their participation for the full 12 months including the exit assessment (Table 1). Three participants requested deletion of their data after completing the study; as such, intake data are presented for 4539 total participants and 3332 completers. The sample that completed the intake assessment met our pre-set targets for representation across categories defined by severity of depression symptoms, age, sex at birth, and self-reported race and ethnicity.

**Table 1:** Participant characteristics in the DMHS

		All (% of total)		*N* completed (completion rate)		*p* value (*χ*^2^ test)
Sample size	*N*	4539	(100.0%)	3332	(73.4%)	*NA*
Sex at birth	Male	1952	(43.0%)	1422	(72.8%)	0.479
	Female	2587	(57.0%)	1910	(73.8%)	
Age	18–25	2030	(44.7%)	1326	(65.3%)	<0.001
	26–45	1426	(31.4%)	1078	(75.6%)	
	46–65	745	(16.4%)	623	(83.6%)	
	>65	338	(7.4%)	305	(90.2%)	
Race/ethnicity	White, non-Hispanic	1443	(31.8%)	1094	(75.8%)	<0.001
	White, Hispanic	1196	(26.3%)	834	(69.7%)	
	Black	667	(14.7%)	472	(70.8%)	
	Asian	1233	(27.2%)	932	(75.6%)	
Depressive severity (PHQ-8 @ screening)	Healthy (0–4)	1132	(24.9%)	904	(79.9%)	<0.001
	Mild (5–9)	1283	(28.3%)	972	(75.8%)	
	Moderate (10–14)	1108	(24.4%)	781	(70.5%)	
	Moderate-severe (15–19)	711	(15.7%)	487	(68.5%)	
	Severe (≥20)	305	(6.7%)	188	(61.6%)	
Diagnoses: depressive disorders	Major Depressive Disorder	1182	(26.0%)	963	(81.5%)	*NA*
	Major Depressive Episode	830	(18.3%)	652	(78.6%)	
	Persistent Depressive Disorder	503	(11.1%)	414	(82.3%)	
	Depressive Disorder, Other	218	(4.8%)	182	(83.5%)	
Diagnoses: anxiety disorders	Generalized Anxiety Disorder	583	(12.8%)	471	(80.8%)	*NA*
	Social Anxiety	545	(12.0%)	424	(77.8%)	
	Panic Disorder	295	(6.5%)	237	(80.3%)	
	Agoraphobia	229	(5.0%)	183	(79.9%)	
Diagnoses: other psychiatric disorders	Obsessive-Compulsive Disorder	511	(11.3%)	398	(77.9%)	*NA*
	Post-Traumatic Stress Disorder	277	(6.1%)	209	(75.5%)	
	Attention-Deficit Hyperactive Disorder	401	(8.8%)	314	(78.3%)	
	Bipolar Disorders	231	(5.1%)	173	(74.9%)	
	Psychotic Disorders	96	(2.1%)	72	(75.0%)	
	Substance Use Disorders	706	(15.6%)	558	(79.0%)	
	No diagnoses	1658	(36.5%)	1398	(84.3%)	

Recruitment bins were designed to achieve a diverse sample with respect to sex at birth, age, race/ethnicity, and depressive symptom severity at screening. Race and ethnicity were based on self-report at screening; participants identifying as Black regardless of Hispanic/Latino ethnicity were classified as Black, all other participants identifying as Hispanic/Latino ethnicity were classified as Hispanic/Latino, participants identifying as Asian and not Hispanic/Latino ethnicity were classified as Asian, and participants identifying as White, unknown or any other race and not Hispanic/Latino ethnicity were classified as Non-Hispanic White or Other. Depressive symptom severity at study entry was assessed using the PHQ-8. Diagnoses were derived using the SAGE-SR (at either of two timepoints when it was administered). To test whether subcategories differed from each other in study completion rate, separate Pearson’s Chi-squared tests for sex at birth, age, race and ethnicity, and depressive severity at screening were conducted (*p*-values indicated in last column, detailed results as follows - effect of sex on completion rate: *χ*2(1) = 0.5008, *p* = 0.479; effect of age on completion rate: *χ*2(3) = 160.39, *p* < 2.2e-16; effect of race/ethnicity on completion rate: *χ*2(3) = 17.947, *p* = 0.000451; effect of intake depression severity on completion rate: *χ*2(4) = 63.039, *p* = 6.658e-13). *PHQ*-*8* Patient Health Questionnaire-8; *SAGE*-*SR* Screening Assessment for Guiding Evaluation-Self-Report.

The sample was selected to span the full range of depression symptom severity at intake, as determined by initial PHQ-8 score (Table 1, Supplementary Fig. 2). Approximately half of the initial sample (47%, *N* = 2124) had a PHQ-8 score ≥ 10, a cutoff value associated with high sensitivity and specificity for capturing DSM-5 depression episodes^23^. Consistent with this expectation, the SAGE-SR suggested a likely depression diagnosis, at either of the two assessment timepoints, for 60.2% of participants. A substantial proportion of participants met criteria for likely anxiety disorders (36.4%) and other psychiatric disorders (48.9%), while 36.5% of participants did not qualify for any DSM-5 diagnosis according to SAGE-SR (Table 1). The completion rate exceeded 60% for all levels of intake depression severity and was significantly higher (*p* < 0.001) among those with an intake PHQ-8 < 10 (77.7%) compared to those with intake PHQ-8 ≥ 10 (68.5%).

### Longitudinal trends in study dropout, assessment completion and protocol adherence

The study enrolled 4539 participants, of whom 3332 completed the full protocol, resulting in an overall retention rate of 73.4%. The proportion of participants who completed the full 12-month study exceeded 65% for all age, sex at birth, and race/ethnicity subgroups. Completion rate showed significant associations with age (range from 65.3% for 18–25 years to 90.2% for >65 years) and race and ethnicity (ranging from 69.7% for White, Hispanic to 75.8% for White, non-Hispanic, Table 1). Student status did not predict dropout rate when controlling for age (*p* = 0.944); however, student status and age were highly collinear, as most of our youngest participants were students.

Socioeconomic factors showed mixed effects on retention among the non-student population. Higher education levels were associated with greater study completion (*p* < 0.001), surpassing 80% for individuals with at least a bachelor’s degree compared to under 70% for those without. However, employment status did not significantly impact retention (*p* = 0.626), as employed and unemployed participants completed the study at comparable rates (77.0% and 80.7%, respectively). Finally, we examined attrition timing and found no significant relationship between participant dropout rates and the schedule of UCLA’s academic quarters (typically ending December, March, and June).

**Fig. 1:**
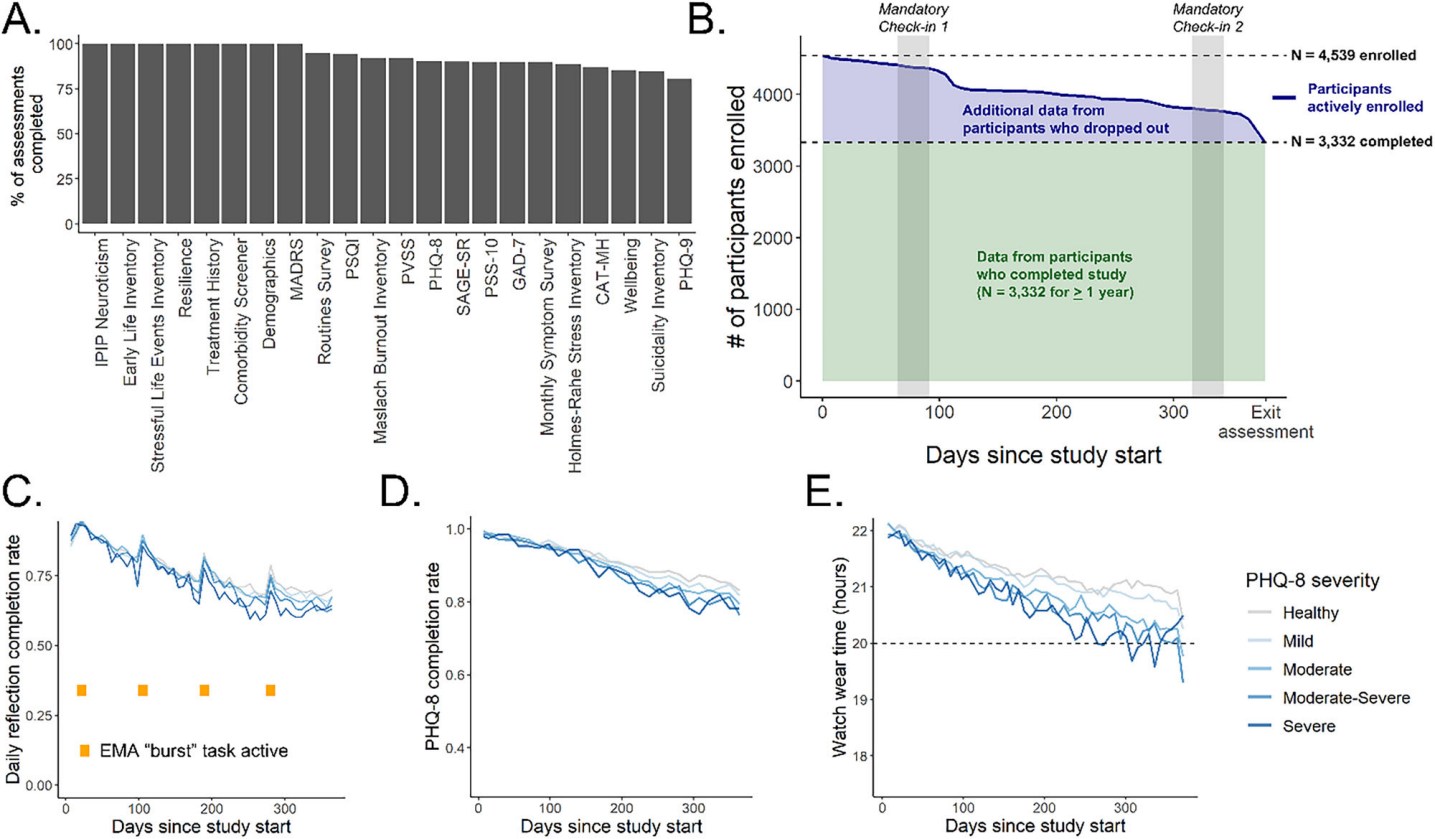
Overview of protocol adherence. **A** Overall task adherence rates for individuals who participated for 12 months (“Completers”). **B** Proportion of participants enrolled across the study duration, separated by depression severity (as measured by the PHQ-8) at screening. Highlighted bar indicates timing of mandatory initial check-in session, after which participants who could not finish the session were excluded from the remainder of the study. **C** Adherence rate for the daily reflection survey task, separated by depression severity. Yellow bars indicate timing of ecological momentary assessment (EMA) “bursts”. **D** Adherence rate for the biweekly PHQ-8 survey separated by depression severity. **E** Mean watch wear time across the study duration, separated by depression severity. Dotted line indicates threshold for daily compliance (20 hours). MADRS: Montgomery-Åsberg Depression Rating Scale (depression); PVSS: Positive Valence Systems Scale (hedonic functioning); PSQI: Pittsburgh Sleep Quality Index (sleep quality); IPIP: International Personality Item Pool (Neuroticism scale); SAGE-SR = Screening Assessment for Guiding Evaluation-Self-Report (psychiatric diagnosis); CAT-MH = Computerized Adaptive Testing-Mental Health (depression, anxiety); Patient Health Questionnaire-8 (depression); PSS-10: Perceived Stress Scale-10 (perceived stress); GAD-7: Generalized Anxiety Disorder 7-item scale (anxiety); PHQ-9: Patient Health Questionnaire-9 (depression).

Increases in dropout rates appeared to cluster around participation milestones, for example, around Month 4, coinciding with the first check-in (weeks 10–13) and the end of the first compensation quarter (Fig. 1B); this check-in was mandatory for continued participation. Dropout rates also increased around the time of the second check-in (weeks 46–49) and before the exit assessment, both of which were required for study completion.

Of the 1207 participants who eventually dropped out, most remained enrolled past the halfway point of the study (median time to drop out of 241 days) (Fig. 1B). For these individuals, we continued to collect data from sensors, self-reports, and clinical interviews until the time that they formally dropped from the study roster. Overall assessment completion rates among those who finished the study were high, exceeding 85% for most tasks (Fig. 1A and Supplementary Table 1), including the biweekly PHQ-8 (90.5%). Compliance with the Daily Reflection task (i.e., at least four Daily Reflection tasks completed per week) by study completers declined from a high of 86.9% in week 1 to 67.2% in week 52 (Fig. 1C). Although baseline (BL) depression influenced the levels of such compliance (*χ*^2^(4) = 17.71, *p* = 0.001), these levels remained high throughout the participation period, even for the group with the most severe BL-depression symptoms (86.8% compliance, first 12 weeks; 72.8% overall) when compared to the no-BL-depression group (88.0% compliance, first 12 weeks; 77.4% overall). Daily completion rates were higher on weekdays (67.1%) than weekends (64.2%). Weeks with High Frequency Current Mood EMA bursts (see EMA burst, Fig. 1C) showed a temporary boost in compliance rates, with a smaller carry-over effect.

Compliance with PHQ-8, among study completers (Fig. 1D) was not significantly affected by BL-depression severity (*χ*^2^(4) = 6.71, *p* = 0.152); while compliance rates among this group declined modestly throughout study, they remained above 80% through the final assessment. Mean watch wear time among completers (Fig. 1E) declined slightly over the course of the study and was influenced by BL-depression (F(4, 2485) = 4.98, *p* < 0.001). Among all completers, mean daily watch wear time was 21.0 hours (SD of 2.2 hours). The average completer met the target wear time threshold (≥20 hours for ≥ 4 days of the week) on 87.4% of the study weeks. Among completers, 67.5% maintained this threshold consistently across 90% of weeks they were enrolled in the study.

### Longitudinal symptom trajectories

Repeated biweekly PHQ-8 assessments allowed for granular examination of depression symptom trajectories (Supplementary Fig. 3). Overall, we observed initial improvement in scores during the first three months, followed by stabilization. This pattern was consistent across severity categories, with steeper improvement for higher BL severity. Such “early improvement” has been reported in other longitudinal studies^24–26^. Symptom severity was subsequently relatively stable, at the group level, over the duration of the study. At the individual level, 38.6% of participants with baseline PHQ-8 < 10 reported at least one score > 10 (accompanied by a ≥5-point increase), and 89.1% of those with baseline PHQ-8 ≥ 10 reported at least one score < 10 (accompanied by a ≥5-point decrease).

### Data reliability: correlational analysis across standard measures

To evaluate construct validity and provide an initial assessment of the reliability of survey responses in our study, we performed correlational analyses at both the aggregate scale level and the item level. Where possible, we compared these correlations against published results from prior population studies in the relevant domains.

**Fig. 2:**
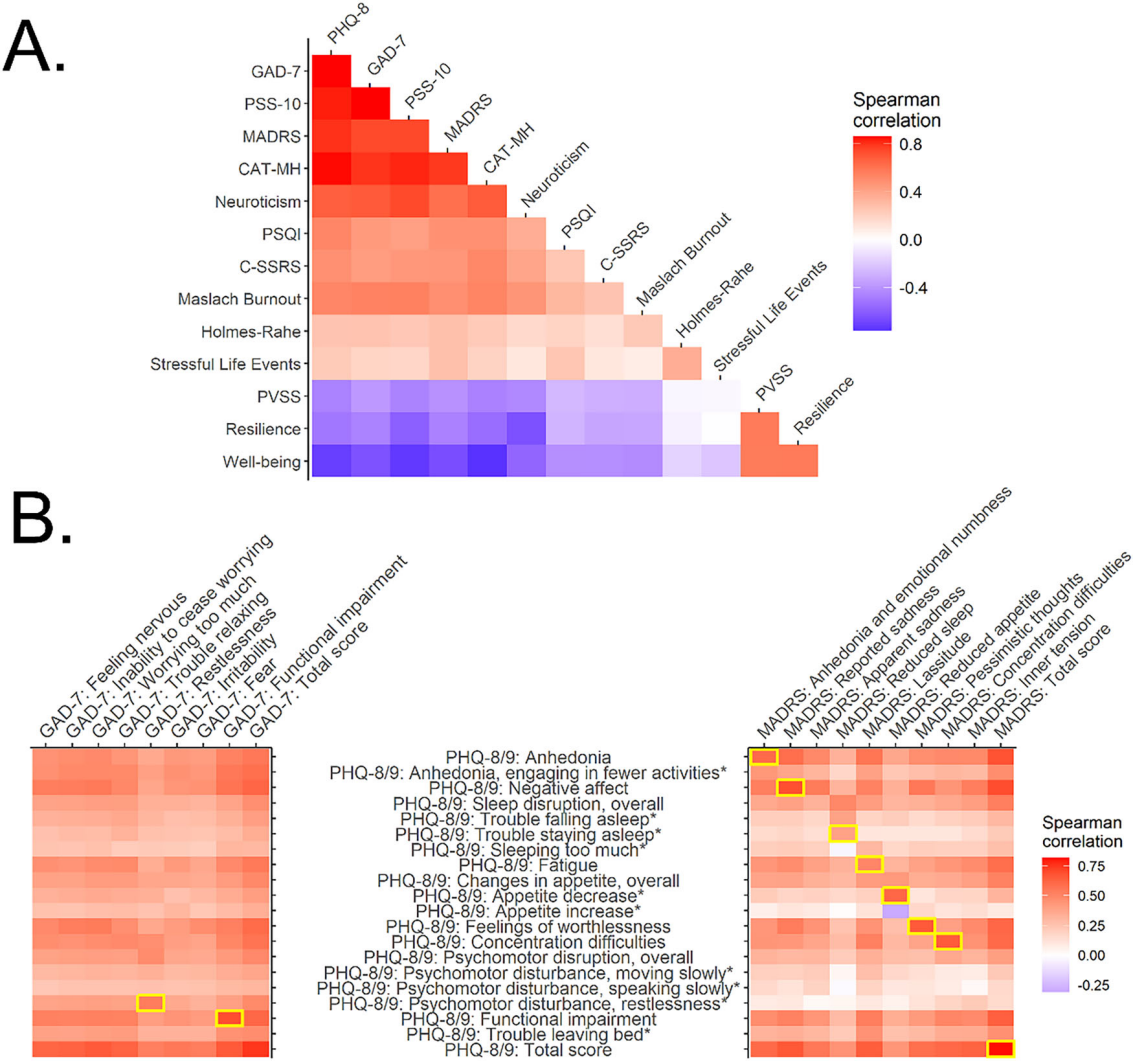
Relationships between scales in the DMHS. **A** Spearman correlations between participant mean scores for all standard assessment scales administered. **B** Spearman correlation between items of the PHQ-8 and GAD-7 at intake (left) and the first timepoint scores of the PHQ-9 and MADRS (right). Highlighted in yellow are pairs of items between the PHQ-8/9 and the GAD-7 and MADRS that assess overlapping symptom constructs. *Indicates items that were added to the modified PHQ-8/9 scale utilized in DMHS. PHQ-8/9: Patient Health Questionnaire-8/9; GAD-7: Generalized Anxiety Disorder 7-item scale; PSS-10: Perceived Stress Scale-10; CAT-MH: Computerized Adaptive Testing-Mental Health, Depressive Severity Score; Neuroticism: neuroticism sub-score of the International Personality Item Pool – Neuroticism, Extraversion, Openness; PSQI: Pittsburgh Sleep Quality Index; C-SSRS: Columbia-Suicide Severity Rating Scale; Maslach Burnout: Maslach Burnout Inventory; Holmes-Rahe: Holmes-Rahe Life Stress Inventory; PVSS: Positive Valence Systems Scale; Resilience: Connor-Davidson Resilience Scale.

At the scale level, we observed strong correlations between several of our core symptom assessment measures (Fig. 2A). At intake, the correlation between total scores of PHQ-8 and GAD-7 was high (Spearman correlation = 0.753), consistent with prior research showing high comorbidity across the domains of depression and anxiety^27^. The correlation between PHQ-9 (self-report) and MADRS (interview-based) conducted on the same day was also high (Spearman correlation = 0.811), in line with prior estimates^28^. Perceived stress was highly correlated with both measures of depression (Spearman correlation = 0.84) and anxiety (Spearman correlation = 0.87), consistent with the hypothesis that such a perception may be a surrogate indicator of disrupted mental health. As expected, we observed inverse correlations between depression, anxiety, and perceived stress scales as compared against scales that assess positive psychological constructs (e.g., wellbeing, resilience, positive valence) (Spearman correlations ranging from −0.38 to −0.72).

To further assess construct validity in our core depression and anxiety instruments (MADRS, PHQ-8/PHQ-9, GAD-7), we conducted a more detailed item-level analysis across these instruments (see Fig. 2B). We found item-level correlations to be substantially lower than correlations at the aggregate scale level, even when comparing the specific subset of items that are directly analogous between scales (e.g. Figure 2B highlighted in yellow). The recent OPTIMUM trial in geriatric depression analyzed item-level correlations between MADRS and PHQ-9 and thus provides a comparator for our study. The correlations between analogous items of the MADRS and PHQ-9 in our study (Spearman correlations ranging from 0.42–0.69, measured at weeks 10–13 of study) are very similar to those observed in the OPTIMUM trial (Spearman correlations ranging from 0.42–0.66 measured at week 10 of the study)^29^. Additionally, both studies found the same four constructs of sadness, feelings of guilt/worthlessness, concentration difficulties, and suicidal thoughts to be most highly correlated across these two instruments, as compared to the remaining items. Discriminant validity was supported by consistently lower correlations between non-analogous item pairs (e.g., PHQ-8 “Trouble staying asleep” with GAD-7: “Feeling nervous”, (median Spearman *r* = 0.337, range = [−0.309, 0.700]) compared to analogous item pairs (median *r* = 0.611, range = [0.386, 0.693]), across both the PHQ-8/GAD-7 and PHQ-9/MADRS comparisons (Fig. 2B).

Overall, the construct validity of our off-the-shelf instruments is comparable to that seen in other large population-based studies in the core domains of depression, anxiety and stress^23,30–32^. This observation suggests that the reliability of participant responses in DMHS was comparable to that of other studies, despite the remote longitudinal data collection. The discrepancies between analogous MADRS and PHQ items seen both in our study and in the literature illustrate the importance of collecting both self-report and interviewer-administered ratings in our protocol.

### Contribution of additional purpose-designed assessments

The custom survey questions we added to our protocol to cover constructs missing from published instruments showed correlations in the moderate range with core depression, anxiety, and stress items (Supplementary Fig. 4). This observation is consistent with our initial expectation that these questions would extract clinical information that is related (i.e. correlated) to core symptomology without being duplicative (i.e. without significant variance) compared to off-the-shelf items.

For items where the DMHS protocol added follow-up questions to disambiguate responses to the PHQ, we observed higher correlations with the analogous items in other scales. For example, the correlation between the MADRS item assessing appetite was higher with our disambiguated version of the analogous PHQ question (Spearman correlation = 0.61) versus the original ambiguous version in the PHQ (Spearman correlation = 0.41). Similarly, the GAD-7 item assessing restlessness had higher correlation with our disambiguated PHQ item (Spearman correlation = 0.54) versus the original ambiguous item on the PHQ (Spearman correlation = 0.48). These findings are consistent with the expectation that adding targeted follow-up questions to disambiguate these responses would improve construct validity of the symptoms being assessed.

### Participant feedback

**Fig. 3:**
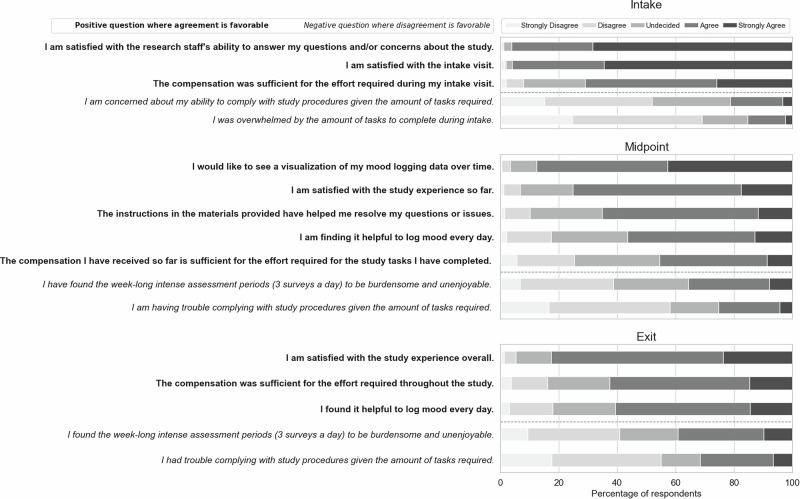
Overview of participant experience feedback at Intake, Midpoint (week 25), and Exit, provided by all participants who were enrolled and finished the survey at the time of administration. Items which are framed in the positive (i.e., agreement is favorable) are indicated with bold text while items framed in the negative (i.e., disagreement is favorable) are indicated with italics.

Feedback surveys at baseline (*N* = 4437), midpoint (week 25, *N* = 3388), and exit (*N* = 3332) indicated that most individuals considered study participation to be a generally positive experience (Fig. 3). At baseline, 96% reported satisfaction with the intake interview, that study tasks were manageable, adequate compensation, confidence in compliance, and satisfaction with staff. Midpoint and exit surveys showed continued satisfaction, perceived benefit of mood logging, no major compliance issues, sufficient compensation, and manageable EMA burden. Visual inspection suggests that participants who dropped out, younger participants, and more depressed participants reported slightly greater difficulty adhering to tasks (Supplementary Figs. 5–7*)*.

Responses to feedback surveys suggest that, beyond finding participation agreeable, most participants perceived benefit in mood logging: 60% agreed to the statement “I found it helpful to log mood every day”. The N = 2021 participants who found logging helpful were asked to choose from among multiple possible reasons for why daily mood logging was helpful to them. 84% of this group reported that it made them more aware of how they are feeling, 50% reported that reflecting on feelings was enjoyable, 10% endorsed a positive impact on responding to challenges, and 9% reported that daily mood logging helped improve mood. Participants were further asked to report on the impact of completing symptom surveys (PHQ-8 and GAD-7) every two weeks; 65% reported that checking in on symptoms improved their mental health, 32% reported that it had no impact, and 3% reported that it decreased their mental health. Finally, participants were asked to indicate, from a list of possibilities, which aspects of the study they found helpful for their mental health. 55% of participants who completed the feedback survey at exit identified daily mood logging as helpful, 44% found completing acute stress prompts on the watch helpful, 27% found the interviewer-administered depression scales helpful, 20% found monthly video journaling helpful, and 19% found the contact with researchers helpful.

## Discussion

The DMHS aims to advance mental health research by demonstrating that data obtained passively from smartphones and smartwatches can provide phenotypes that objectively and precisely characterize physiological, behavioral, and emotional components of depression and anxiety at a large scale. This paper documents the project’s success in completing two foundational steps for achieving this objective. First, we demonstrated the feasibility of enrolling a sample of more than 4,000 individuals representing the full range of depression severity expected in the population and retaining most of them for a full year of intensive, and nearly entirely remote, data collection. Second, we established a protocol for obtaining a sufficiently broad range of digital phenotypes and symptomatic measures to capture the complexity and heterogeneity of depression and anxiety, and their relationship to both participants’ experience of stressful events and their subjective sense of their ability to cope with their life circumstances and events. The DMHS sample was more diverse (by age, self-described race, and ethnicity) and displayed a greater balance of males and females than previous mental health digital sensing studies^33–35^. Most participants across these subgroups adhered to the assessment protocol throughout their participation in the study; we therefore anticipate that results from future DMHS analyses will be broadly generalizable.

Recruitment and retention of participants have been challenging for longitudinal studies of remote digital assessment in mental health, even those with a relatively limited burden of active assessment^36^. The high proportion of participants who completed the full DMHS protocol (about 73% of the overall sample) is therefore noteworthy, given the intensive commitment it required (37 hours over the 12 months). Additionally, most participants who did not fulfill the requirements to be considered study completers still provided both sensor and survey data for nearly the entire study period before they dropped out. Therefore, these individuals will contribute to virtually all future analyses aimed at assessing associations between digital phenotypes and self-reported symptoms. As in previous digital mental health studies, retention rates were lower among younger participants and members of ethnic minority populations^37,38^; however, these decrements were relatively small.

We have considered several possible factors that could explain the relatively high retention rate in the DMHS compared to other studies: (1) our employment of devices considered by participants to be user-friendly (iPhones, which most already owned, and Apple Watches); (2) manageable study burden and adequate compensation; (3) the possible perceived benefit of daily mood logging may have been a motivating factor to stay engaged in the study, given the finding that 60% of participants responded that they found it helpful to log mood every day; and (4) a protocol that involved study team members proactively monitoring daily data completion and communicating regularly with participants. Regarding this last factor, the protocol included several features that facilitated participants staying on track to provide data at the appropriate times. We implemented automated procedures to detect data missingness, for example daily data compliance reporting with automated reach-out flags; these procedures enabled staff to contact participants with a fast turnaround time, so that they could complete a missing task within the expected time range. Additionally, participants were able to track data completion through a user-friendly interface in the Research app. They also received an automated quarterly progress email with a summary of task completion and compensation earned. Finally, the protocol provided multiple opportunities for participants to speak directly with study staff. For example, during assessment sessions, study staff conducted 10-minute semi-structured conversations with participants prior to administering rating scales. Such interactions provided participants an opportunity to check in with staff about all aspects of their study participation and share feedback about their experience. The above factors, as reported through direct participant feedback, suggest that relatively straightforward approaches can be used to sustain participant engagement even with extended and intensive assessment protocols. However, more systematic exploration of these and other potential factors influencing acceptance of longitudinal digital sensing will be necessary for this form of assessment to achieve integration in mental health care. From this standpoint, the high retention rate seen in DMHS participants who fulfill lifetime criteria for severe mental health diagnoses (including bipolar disorder and psychotic disorders) is highly encouraging.

The DMHS differs from prior digital phenotyping studies of mental health in its size and duration; the wide range of digital sensors assayed, including sensor types (e.g., those passively assessing facial expressions with high sampling frequency), which have not previously been employed in this field; the frequency and extensiveness of symptom assessments (by self-report scales, EMAs, and interviews); and the inclusion of purpose-designed questions not asked in standard surveys. We hypothesized that our strategy of selecting measures by systematically mapping survey items onto a unified inventory of mental health constructs could enable coverage of relevant domains while minimizing redundancies. The analyses that we undertook to evaluate correlations between different surveys suggest that we succeeded in achieving this initial objective; instrument-level correlations were high, but the lower level of correlation between individual items supports our view that the battery enabled measurement of many distinct constructs to associate with digital sensing features. This assessment battery required a substantial time commitment from participants that would likely not be feasible in efforts to further scale up mental health digital phenotyping studies. We therefore anticipate that a key aspect of DMHS analyses will be determination of the degree to which digital sensing data predict scores across different instruments; the results of these analyses may suggest the optimal approach for streamlining batteries for future studies.

Surprisingly few studies have conducted longitudinal evaluations of variability in depression and anxiety symptoms outside of mental health treatment settings; studies that have examined this topic have mostly been of short duration and with infrequent assessments^39^. The 12-month duration of the DMHS offered the opportunity to detect clinically meaningful changes in symptomatology in a substantial proportion of participants. It is possible to time such major shifts in clinical state with a high degree of precision, as we obtained PHQ-8 and GAD-7 assessments biweekly throughout the study; analyses of the temporal relation between digital sensing features and major shifts in clinical state can thus be conducted in the DMHS with a high degree of precision. Previous studies suggest, for example, that one possible mechanism underlying our observation of initial improvement followed by stabilization of depression scores could be that individuals have a greater propensity to enroll in research studies during periods when symptoms are more severe, and that after enrollment the distribution of symptom severity follows a regression to the mean^40,41^. Quantifying reliable, clinically meaningful changes in depressive state is inherently complex, particularly within the context of a multimodal observational study combining several distinct approaches to measuring depression symptoms. Given these nuances, a more detailed analysis of these longitudinal symptom trajectories and their relation to digital phenotypes will be the focus of future papers.

Among those participants who completed the full 12-month study, most continued to display a high degree of adherence to study tasks throughout the duration of their participation. Furthermore, the rate of completing these tasks was similar across the different modalities (surveys administered via REDCap, surveys administered via the Research app, and watch wear, Fig. 1). The high completion rates across active and passive assessment modalities, in individuals across a broad range of diagnoses and symptom severities, suggest that patients may be able and willing to adhere to care plans that involve extended watch wear and data sharing. The decrease in adherence rate of daily mood EMA over the course of the study was greater than for other data types (although nearly half of participants continued to be adherent with these assessments on day 364), suggesting the utility of exploring alternative protocols for collecting this type of data. In designing such protocols, it is important to note that most participants reported that they considered it useful and non-burdensome to provide mood logging data.

Periodic assessment of participant attitudes towards digital assessments was an important component of the DMHS design. Most individuals reported, consistently, that participation was a positive experience, suggesting that research studies using extensive digital phenotyping may be broadly acceptable to potential participants.

Several limitations should be considered. First, while the study sample was highly diverse with respect to multiple demographic and clinical variables, it did not attempt to achieve diversity in socioeconomic measures that may be important for both understanding depression and anxiety trajectories and usage patterns of digital devices; our use of approaches that depended on the internet may have limited our recruitment of individuals most affected by the socioeconomic “digital divide” in device usage^42^. Additionally, all participants were recruited from the populations served by a single healthcare system and university, in a location (coastal Southern California) with minimal seasonal climate variation. This fact potentially limits the generalizability of findings related to behaviors (e.g., physical activity) that could be strongly affected by such seasonal variation. It is therefore essential that future studies attempt to replicate findings from DMHS in more diverse locations. Second, study participation was additionally limited to Apple iPhone users (or those willing to use a loaner iPhone) who may differ in unknown ways from individuals who use other devices. We note that only about 2% of study participants received a loaner iPhone, and therefore it is unlikely that any differences between those who used such a loaner device and other participants could meaningfully influence any results of the study. Third, we do not yet have an adequate understanding of the factors responsible for participants dropping out of the study, and therefore additional information is needed to design effective strategies to assess and then mitigate these factors. Finally, while we have detailed data about changes in symptomatology across the duration of the study, we did not systematically attempt to identify factors outside of the study that could contribute to such variation.

In summary, the DMHS represents a unique dataset, leveraging widely available and user-friendly consumer devices to demonstrate the feasibility of large-scale digital phenotyping and advance our understanding of common mental health conditions. Although analyses of this dataset are ongoing, we anticipate that they will demonstrate novel associations between a wide array of digital phenotype domains (including detailed assays of movement, sleep, social interaction, speech features, facial expressions, keyboard dynamics, and sentiment) and a set of symptomatic assessments that were more extensive and more frequently obtained than those in previous digital mental health studies. These association results may themselves contribute to more precise and objective frameworks for characterizing mental health symptoms and for unraveling the complexity and heterogeneity of mental health disorders. The results will also encourage further studies that will use phenotypes from digital devices to achieve even more ambitious aims. For example, the heterogeneity of depression and anxiety has presented an obstacle to efforts to identify specific genetic variants contributing to these conditions^43–45^. Our experience with the DMHS indicates that it should be feasible to remotely obtain precisely defined and objective digital phenotypes for the much larger samples that would be needed for such genetic discovery studies. Similarly, we anticipate that DMHS results will suggest immediate strategies for studies assessing the value of including digital phenotypes in clinical decision making and other precision treatment efforts.

## Methods

The methods described below are for the main (12-month) DMHS. The DMHS protocol was informed by insights from two pilot studies (DMHS Pilot 1 [DMHS-P1] and DMHS Pilot 2 [DMHS-P2]; described in final sub-section below).

### Participant characteristics

To obtain a sample of 3000 participants completing the study and maintaining minimal protocol adherence, we set an overall recruitment target of 4500 individuals, based on anticipated attrition over the 12-month study duration. The target (*N* = 3000) was selected to support subset analyses of participant cells, each defined by four criteria (five symptom severity levels, two sexes [at birth], four age groups, and four self-described ethnicities), with a mean of 18 per cell. This target was distributed as follows: (1) 1000 students enrolled at the University of California Los Angeles (UCLA); (2) 2000 patients receiving care within the UCLA Health system. The student cohort was intended to enable comparison to other digital mental health studies of college students and constitute a more homogeneous sample for evaluating signal strength, while the UCLA Health cohort was intended to enable broader analysis of demographic groups and daily routines. Additionally, we aimed to include within the 3000-participant sample, 200 peripartum individuals recruited from either the UCLA student or health system populations.

To enable analyses across the full range of severity of depression symptoms, recruitment was stratified by depression symptom severity, as measured by the Patient Health Questionnaire-8 [PHQ-8]^46^ administered during online screening. Target composition was 28% with minimal or no depressive symptoms (PHQ-8 < 5), 28% mild (PHQ-8 5–9), 24% moderate (PHQ-8 10–14), and 13% with moderate-severe (PHQ-8 15–19) and 7% severe symptoms (PHQ-8 ≥ 20).

The recruitment targets for these “severity bins” were balanced for sex at birth, age, and ethnicity, such that the demographic distribution was consistent across severity levels. We sought to recruit evenly by male and female sex at birth, except for the peripartum cohort. Within the UCLA Health sample, recruitment was stratified by 4 age categories 18–25, 26–45, 46–65, and over 65, where we aimed for a balanced distribution from the three younger categories and at least 15% over 65. Regarding self-reported race and ethnicity, we aimed to collect a minimum of 15% in each of four subgroups. With a goal of making the distribution as even as possible within the recruiting time window, the targets doubled the population prevalence of three of the subgroups and capped the most prevalent group. The resulting target distribution was as follows: From among the UCLA Health cohort, we aimed to recruit a sample of at most 45% Non-Hispanic White or Other and at least 21%, 18%, and 16% Asian, Hispanic, and Black, respectively. Among the UCLA students, our target was at most 40% Non-Hispanic White or Other, and at least 30%, 15%, and 15%, Asian, Hispanic, and Black, respectively.

Other eligibility requirements are detailed in Supplementary Table 2. Briefly, participation required willingness to use a study-provided Apple Watch for the duration of the study and ownership of an eligible iPhone. A few individuals (*N* = 70) participated using a loaner iPhone; these participants were eager to participate, met all other eligibility criteria, and either didn’t own a suitable iPhone or lost or broke their personal iPhone after enrollment and were unable to replace it during the study period. We considered it inequitable to exclude these individuals. All participants were responsible for their own data plans while participating in the study.

### Participant recruitment and enrollment

Recruitment employed a multifaceted approach, including targeted messages through the UCLA Health patient portal (allowing for stratification based on demographics and clinic affiliation, with a focus on increasing enrollment of peripartum individuals); automated telephone recruitment to UCLA Health System patients; and communications via the UCLA Student Registrar’s office, departmental email lists, campus organizations, social media, email newsletters, and a prospective participant registry. All materials directed individuals to a study website providing details on eligibility, time commitment, and compensation. Prospective participants initiated the process of screening for eligibility via the website. Online screening consisted of a questionnaire assessing key eligibility criteria (age ≥18, English fluency, compatible iPhone or willingness to use study-provided phone), self-reported demographics, and the PHQ-8 for depression severity stratification. Complete eligibility criteria are detailed in Supplementary Table 2. Eligible individuals were presented with an online informed consent form to review and sign electronically. Prospective participants could additionally contact our study team by email or phone prior to initiating online screening, prior to enrolling, or at any time during study participation.

### Participant safety and ethical considerations

The study was conducted in accordance with the Declaration of Helsinki and relevant institutional guidelines and regulations. Ethical approval was obtained from the University of California, Los Angeles Institutional Review Board (DMHS-P1: #20-000995; DMHS-P2: #20-002337; DMHS main study: #21-001558). All participants provided written informed consent electronically prior to participation. A safety protocol, overseen by licensed clinical staff, was implemented to follow up on any disclosures of suicidality in clinical interviews or self-report surveys. Clinical trial number: not applicable.

### Device provision and data collection

To obtain survey and digital sensing data, we mailed all enrolled participants a study kit prior to their intake assessment, containing an Apple Watch, a participant instructions packet, and – for those without a compatible device – a loaner iPhone. During the remote intake assessment, study staff guided participants through device setup and enrollment in the Apple Research app^47^, which managed the collection of sensor data, device data, and surveys. The app is a platform that enables individuals to securely share data from their devices for research studies, preserving their privacy. Participants were provided with detailed information about all data types to be collected and were required to authorize sharing for each type. They could review and manage their data-sharing permissions within the app at any time throughout the study. Data collection spanned 52 weeks, utilizing the provided devices, the Apple Research app on participants’ iPhones, online surveys, and assessment sessions with study staff. Participants were instructed to wear the Apple Watch for at least 20 hours daily, including during sleep, and to charge it for at least two hours daily while it was connected to their iPhone and Wi-Fi.

Intake assessment sessions were conducted upon enrollment by research coordinators who were trained to follow an intake protocol, and adherence was monitored by the Project Director. During these sessions, study staff confirmed the iPhone iOS version, paired the Apple Watch, guided participants through Apple Research app enrollment, reviewed the study schedule, reiterated device charging requirements, explained compensation, and emphasized ongoing data monitoring. Check-in assessment sessions, including interviewer-administered rating scales, were conducted remotely or in-person, occurring at approximately weeks 10–13 and 46–49. Participation concluded with an exit assessment. Participants could track completion of active tasks within the Apple Research app itself. The Apple Research app did not provide participants with real-time feedback or visualizations based on their mood entries, to minimize potential reactivity effects. Feedback was limited to quarterly progress report emails summarizing average daily mood ratings and compensation earned.

Interviewer-administered rating scales were obtained using Research Electronic Data Capture (REDCap), a platform that was also used to collect a subset of the self-report surveys. Surveys that were collected at high frequency (as detailed below) were administered using the Apple Research app installed on participants’ iPhones; each survey had its own schedule for task reminders.

### Assessment protocol

**Fig. 4:**
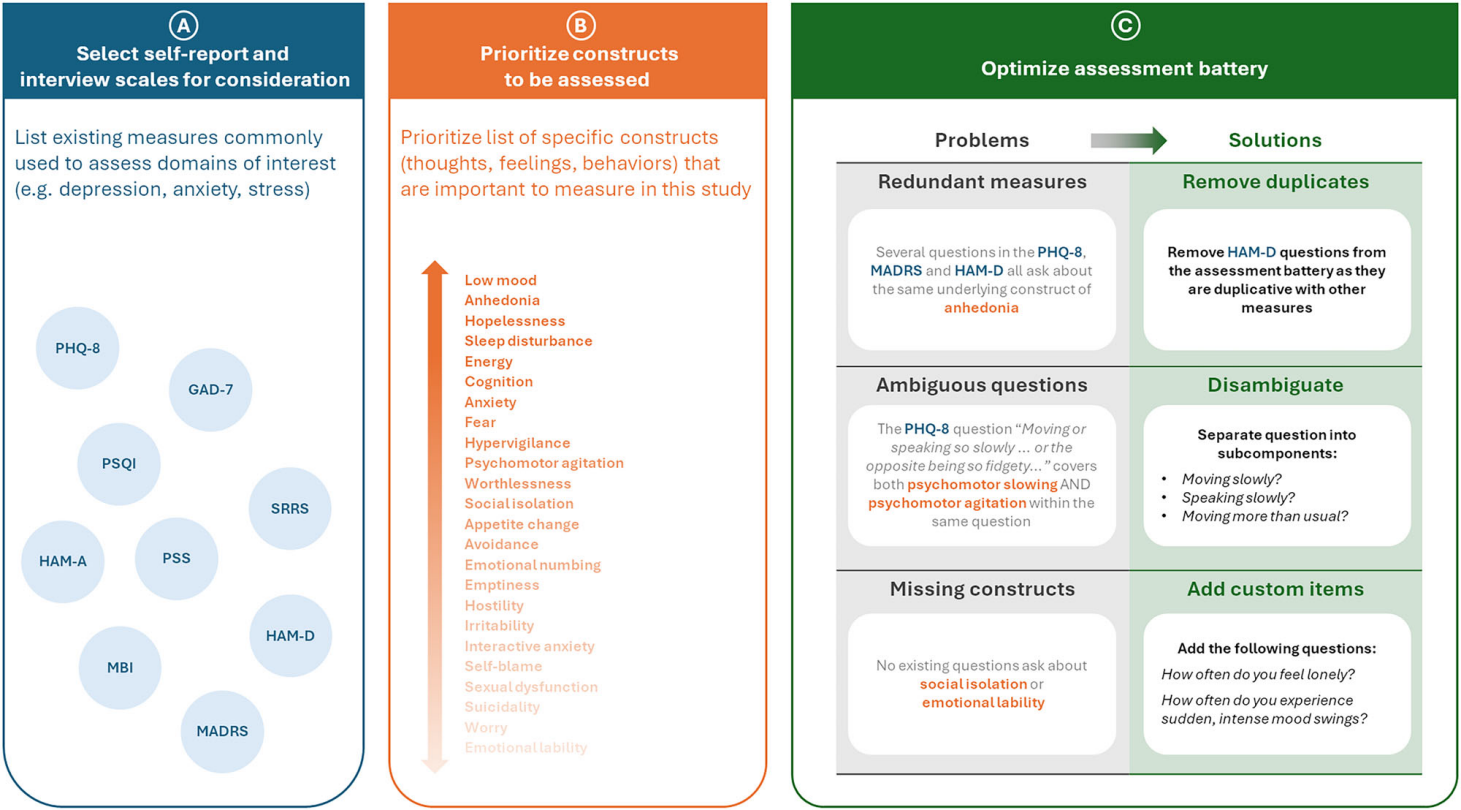
Schematic diagram of the three-step process used for developing the Digital Mental Health Study (DMHS) main study assessment protocol. **A** We first selected for consideration a set of widely used validated interviews and self-report scales (key to abbreviations and citations below). **B** We developed a separate prioritized inventory of mental health constructs (signs, symptoms, or other phenomena related to mental health), drawing on recent field-wide classification systems (HiTOP, RDoC and DSM-5). **C** Each question in the potential instruments from Step A was then mapped to the construct from Step B which it covered. At this point, we could determine which constructs were receiving redundant assessment (i.e., assessed by multiple questions across different potential instruments) and which represented a gap in coverage (i.e., not assessed by any question across these instruments). We optimized the assessment protocol by minimizing redundant coverage, increasing the specificity of questions with ambiguous construct mappings, and adding sets of custom assessments to fill gaps in coverage. For example, the HAM-D showed similar overall construct coverage to the MADRS but with less specificity in each item, and therefore HAM-D was dropped, with the MADRS becoming the primary interview-based depression assessment. CAT-MH: Computerized Adaptive Testing for Mental Health; GAD-7: Generalized Anxiety Disorder 7-item scale; MADRS: Montgomery-Åsberg Depression Rating Scale; SCID: Structured Clinical Interview for DSM Disorders; HAM-D: Hamilton Depression Rating Scale; HAM-A: Hamilton Anxiety Rating Scale; HiTOP: Hierarchical Taxonomy of Psychopathology; RDoC: Research Domain Criteria; DSM: Diagnostic and Statistical Manual of Mental Disorders.

We aimed to implement a protocol that would enable elucidation of the relationship between a broad range of digital phenotypes and traditional assessments of depression and anxiety, obtained by interviewer-administered rating scales and self-report questionnaires. We therefore optimized a protocol to most fully capture the complexity of these conditions, and to collect information about both exposure to stressful events and subjective experience of stress, without creating an unacceptable burden for participants. The design of the symptom assessment protocol followed a three-step process (Fig. 4) with the goal of comprehensively evaluating core constructs while reducing participant burden by limiting item redundancy. First, we identified a broad pool of existing validated surveys and scales relevant to mental health assessment (Fig. 4A). Concurrently, we compiled a separate and independent inventory of mental health “constructs”, encompassing symptoms and other phenomena associated with depression and anxiety (Fig. 4B). This inventory drew upon prior work in three established frameworks: the Hierarchical Taxonomy of Psychopathology (HiTOP)^48^, the National Institute of Mental Health’s Research Domain Criteria (RDoC)^49^, and the DSM-5 ^7^. We prioritized a list of specific constructs for this assessment protocol based on our consensus regarding their relevance to the aims and objectives of the study.

Team members with expertise in depression and anxiety research then mapped individual questions from the above pool of survey instruments to the list of prioritized constructs. For example, the eighth item of the PHQ-8 (a query of “moving or speaking so slowly that other people could have noticed, or the opposite – being so fidgety or restless that you have been moving around a lot more than usual”) mapped to three distinct constructs: “psychomotor slowing: movement”, “psychomotor slowing: speech”, and “psychomotor agitation”. This comprehensive mapping allowed us to evaluate construct coverage across multiple potential instruments and to address three key issues. First, it revealed redundancies, suggesting assessments or items that could be removed to reduce burden without compromising essential information. Second, it identified survey questions that required disambiguation to achieve the specificity necessary for achieving study goals. Finally, it identified gaps in coverage of constructs that we deemed important to study goals but that were not assessed by existing instruments.

We leveraged this information in a final optimization step, employing a three-pronged approach (Fig. 4C). First, we eliminated redundant instruments, prioritizing those with the clearest and most specific questions when constructs were covered by multiple instruments. Second, we supplemented existing questions with custom follow-up questions to disambiguate construct mappings. For example, we supplemented our version of the PHQ-8 by adding contingent questions to differentiate psychomotor agitation from psychomotor slowing of movement and psychomotor slowing of speech. Third, we added purpose-designed questions to address identified gaps (see Monthly Symptoms and Events section below), ensuring that all essential constructs were assessed (descriptions of all survey instruments are in Supplementary Table 3).

**Fig. 5:**
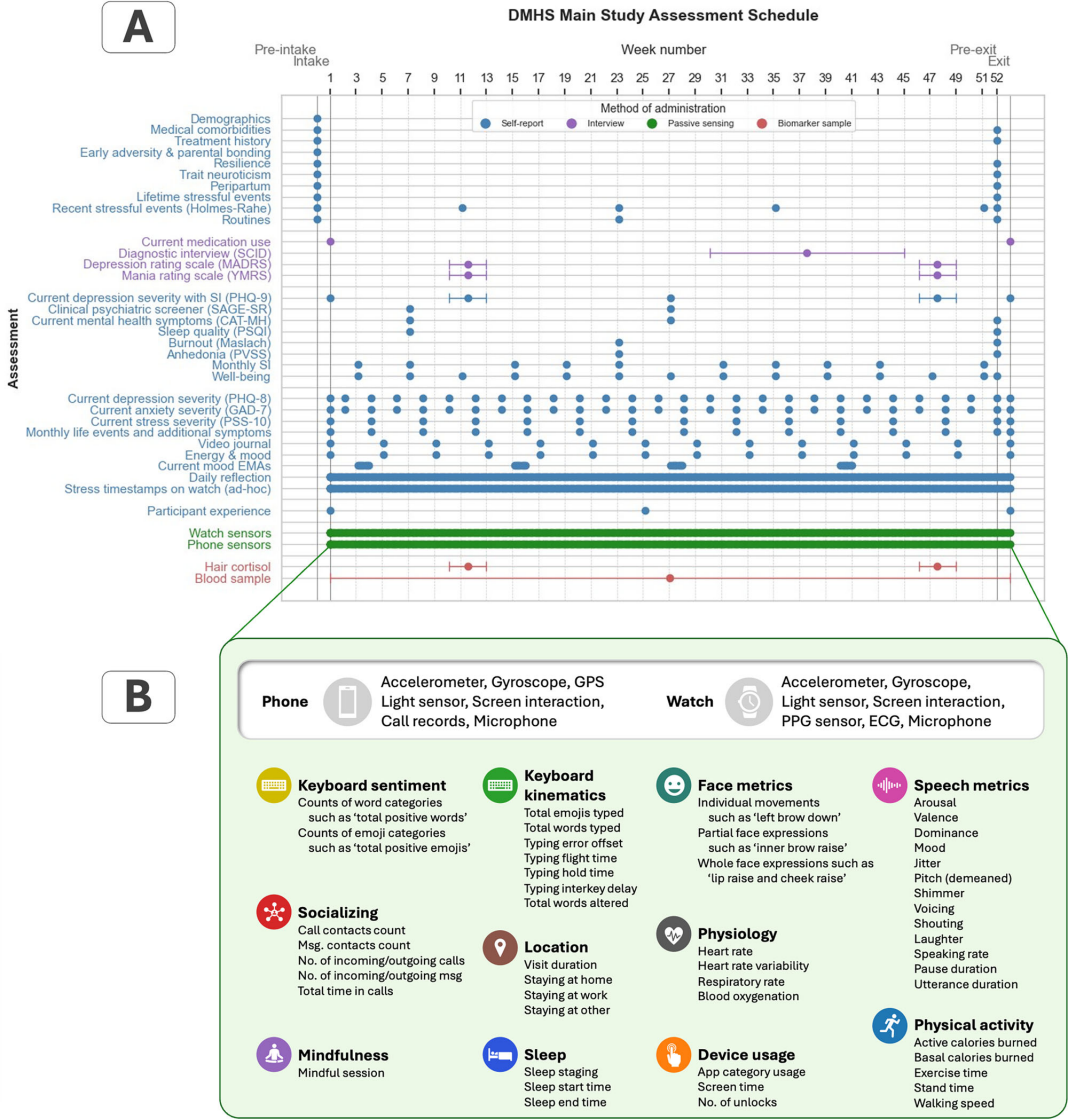
Overview of the final assessment protocol implemented in the DMHS. **A** Depiction of the protocol, which spans a period of at least one year for every participant completing the study and encompasses a range of assessments including self-report measures, interviews conducted by trained staff, and continuous digital sensor streams (from iPhone and Apple Watch). **B** Summary of key features collected in the passive data monitoring protocol, derived from the combined set of Apple Watch and iPhone data sensors. These features leveraged the Apple SensorKit and HealthKit data collection platforms. MADRS: Montgomery-Åsberg Depression Rating Scale; YMRS: Young Mania Rating Scale; SCID: Structured Clinical Interview for DSM Disorders; SAGE-SR = Screening Assessment for Guiding Evaluation-Self-Report; CAT-MH: Computerized Adaptive Testing for Mental Health; PHQ-9: Patient Health Questionnaire-9; PSQI: Pittsburgh Sleep Quality Index; Maslach Burnout: Maslach Burnout Inventory; PVSS: Positive Valence Systems Scale; Holmes-Rahe: Holmes-Rahe Life Stress Inventory; PHQ-8: Patient Health Questionnaire-8; GAD-7: Generalized Anxiety Disorder 7-item scale; PSS-10: Perceived Stress Scale-10.

In establishing the final protocol (Fig. 5), we selected the cadence for each survey based on expected symptom variability. For example, subjective experience of stress and current mood, given their potential for intra-day variability, were evaluated up to multiple times daily via ecological momentary assessment (EMA). We evaluated self-report symptoms of depression and anxiety biweekly throughout the 52-week study. Variables that we expected to be relatively stable across the course of a year were measured less frequently (e.g., occurrence of stressful life events was assessed quarterly). The final study protocol encompassed self-report questionnaires, interviewer-administered rating scales, and collection of hair samples for potential measures of long-term hormonal stress response (details of study assessments are provided below). The estimated total time commitment for these assessments was approximately 37 hours over the 52 weeks. Each of these assessments is described in further detail below.

We assessed depression and anxiety symptoms biweekly using the PHQ-8 and the Generalized Anxiety Disorder 7-item scale (GAD-7)^31^, respectively. Both surveys were administered remotely via the Apple Research app (except when administered via REDCap at the exit assessment). The full PHQ-9 (which includes a question on suicidality) was collected quarterly via REDCap. To disambiguate responses relating to psychomotor, sleep, appetite, and anhedonia constructs, we added contingent questions to both the PHQ-8 and PHQ-9 such that, if participants endorsed one of these items, they were asked to further clarify which specific symptoms they were experiencing (see Supplementary Table 4A).

To obtain a more comprehensive assessment of depression symptomatology than that obtained from these self-report surveys, trained raters administered the Montgomery-Åsberg Depression Rating Scale (MADRS)^50^ at two timepoints (approximately weeks 10–13 and 46–49), always on the same day that a PHQ-9 was obtained. Raters were research coordinators and advanced Psychology graduate students who were trained to administer these clinical rating scales. Training involved reading the rating scale manuals, attending training seminars and reviewing recordings of previous administrations. In addition, raters were required to meet thresholds for inter-rater reliability (i.e., their ratings on practice assessments were consistent with gold standard ratings established by experts) as well as certain thresholds for both interviewing skills and diagnostic competency. Interviews were reviewed in weekly clinical supervision meetings by our diagnostic assessment supervisor. Raters did not have access to participants’ self-reported symptom scores prior to the interview. While both self-report and interviewer-administered rating scales represent standard, widely utilized methods of symptom assessment, results from these different modalities do not consistently demonstrate high correlations^32^. Each instrument may preferentially capture distinct features of depression; self-report scales such as the PHQ-9 emphasize assessment of an individual’s subjective experience of symptoms and emotional state, whereas interviewer-administered rating scales like the MADRS may better capture observable behaviors and symptoms that could be difficult for participants to accurately self-report. We chose to obtain these scales around the end of the 1^st^ quarter and middle of the 4th quarter (rather than at intake and exit) to ensure that we could assess associations of these scales to sensor data collected both before and after the assessments.

A customized survey was administered that incorporated concepts from existing scales^38,51^ with the aim of distinguishing between passive and active suicidal ideation over the previous two weeks. This self-report assessment was administered every four weeks throughout the study via REDCap. In addition to providing research data, this survey identified participants with active suicidal ideation who warranted a follow-up assessment (see Participant Safety and Ethical Considerations, above).

Perceived stress was captured every four weeks via the Apple Research app using the Perceived Stress Scale-10 (PSS-10), which measures subjective appraisal of whether one’s life circumstances and events exceed one’s adaptive capacity^52^. In addition, participants were asked to rate whether they felt stressed, as part of the Daily Reflection survey, and could record a Stress Log via the Apple Watch at any point during the study (Supplementary Tables 3 and 5A).

Early life adversity was assessed once using an Early Life Adversity and Parental Bonding questionnaire at the beginning of the study. Exposure to major stressful life events, which are typically considered moderators or predictors of mental health states^53,54^, was assessed using a Lifetime History of Stressful Life Events survey (adapted from the List of Threatening Experiences^55^, Stressful Life Events Screening Questionnaire^56^ and the Life Events Checklist for DSM-5^57^) at the beginning and end of the study via REDCap; this survey was supplemented on a quarterly basis by the Holmes-Rahe Life Stress Inventory^58^.

To assess symptoms relevant to depression and anxiety not measured in the surveys described above, we developed a custom survey consisting of 13 questions (some of which had follow-up queries) which we asked participants to answer every four weeks throughout the study via the Apple Research app (Supplementary Table 4B). To develop this custom survey, we identified all concepts in the construct inventory (Fig. 4B) that did not map to any of the surveys that we were considering using. Then, three experts on depression and anxiety (ZDC, MGC and NF) ranked these concepts based on their judgment of their relevance to the study aims; those that demonstrated the highest degree of consensus between the three raters were selected for inclusion in the study.

We assessed participants for a broad range of mental health conditions beyond depression and anxiety. The Computerized Adaptive Testing-Mental Health survey (CAT-MH)^59^ is a self-report instrument that uses an adaptive algorithm to provide a rapid screen for current symptoms of depression, anxiety, mania/hypomania, post-traumatic stress disorder, and substance use disorders. We obtained this survey from all participants at weeks 7 and 27, and at exit via REDCap. Diagnostic information for major mental health disorders was obtained from all participants (at weeks 7 and 27) through completion of a comprehensive self-report behavioral health diagnostic screening tool, the Screening Assessment for Guiding Evaluation: Self-Report (SAGE-SR)^60^. All individuals who endorsed symptoms suggestive of mania or hypomania on the SAGE-SR or CAT-MH were further assessed for these conditions by a trained rater using the Young Mania Rating Scale (YMRS) at approximately weeks 10–13 and 46–49^61^.

We invited 240 participants for a structured diagnostic interview to confirm diagnosis of a current major depressive episode (MDE), selected based on inconsistent screening scores on the PHQ-8 (at week 26), PHQ-9, CAT-MH, and SAGE-SR (all at week 27). These NetSCID interviews, a computerized version of the Structured Clinical Interview for the DSM-5 (SCID-5)^62^, were administered by trained and reliability-certified raters (research coordinators and advanced Psychology graduate students).

EMAs, representing subjective ratings of emotional states by participants while in their natural environment, may more accurately represent those states than the responses to retrospective self-report scales. We obtained EMAs using two schedules. Participants completed a Daily Reflection EMA via the Apple Research app throughout their participation in the study, rating overall mood over the past 24 hours: sadness, anxiety, stress, calm, energy, happiness (Supplementary Table 5A). Additionally, we obtained High Frequency Current Mood EMA “bursts” on a quarterly basis; these bursts consisted of self-ratings of immediate mood three times daily for seven consecutive days (Supplementary Table 5A).

Participants completed self-report surveys capturing their feedback about their experience in the study at the beginning, middle, and end of the study.

To further develop metrics correlating mood state with facial and speech features, participants provided monthly video recordings via the Apple Research app on their iPhones. These recordings captured responses to self-administered questions probing recent emotional state, including general mood, instances of anger, stress, and sadness, as well as recent positive events (Supplementary Table 5B).

10-minute semi-structured conversations with the study staff were collected, with consent from both the participant and the study staff. These were collected twice, immediately prior to the MADRS, at approximately weeks 10–13 and 46–49. The conversational prompts asked by raters are listed in Supplementary Table 5C. In addition, all clinical interviews were AV recorded for safety and quality purposes, and participants could opt to have their recordings used for the study.

Positive aspects of mental health were assessed monthly via the Apple Research app using a brief 4-item Wellbeing survey adapted from the Flourishing Scale and the Multidimensional Assessment of Interoceptive Awareness (MAIA). Participants reflected on the past month with respect to positive affect, supportive social relationships, sense of life purpose, and interoceptive awareness of emotional state (Supplementary Table 5D).

Participants were offered the opportunity to provide a blood sample for future genetic analyses at any time during the study for additional compensation. A subset of participants was offered the opportunity to provide hair samples at an in-person check-in session, at approximately weeks 10–13 and 46–49, for additional compensation. Hair samples were collected by study staff to assay cortisol levels.

### Passive Digital Assessments

The DMHS employed an extensive passive data collection protocol (Supplementary Table 6), leveraging the capabilities of Apple’s HealthKit and SensorKit platforms to unobtrusively assess participants’ physiology, behavior, and emotional expression in daily life. HealthKit and SensorKit are data collection frameworks that grant access to a broad range of data streams from both iPhone and Apple Watch, after the participant authorizes sharing the data with the study. These frameworks employ on-device processing for many data streams to protect privacy. For example, rather than transmitting the raw GPS coordinates that have been used in prior digital phenotyping research, SensorKit algorithms categorize a person’s location directly on the phone and only report movements between anonymized locations. The raw location of participants is never shared or transmitted from either device.

The study protocol made use of many of the sensors on these devices (Fig. 5B). Sensor data included accelerometer, gyroscope, and ambient light sensor readings from both iPhone and Apple Watch. Additionally, iPhone data included microphone input sampling (processed on-device for extraction of speech features such as pitch), screen time, call and text messaging metadata (such as duration and counts, excluding content of the calls), and application usage metadata (such as total duration of categories of apps). The Apple Watch also provided microphone input (similarly processed on-device), screen time, photoplethysmography (PPG) sensor data, and opportunistic electrocardiogram (ECG) sensor data.

From these diverse sensor data, over 200 behavioral and physiologic features were extracted via HealthKit and SensorKit, summarized here (see Supplementary Table 6 for details).

Mobility and location features, derived from iPhone location data, included visit duration at various anonymized locations and the total amount of time spent at home and work. Excluded GPS data and specific location information. Sleep-related features, such as sleep staging (REM, light, deep), sleep start time, and sleep end time, were derived using the Apple Watch’s on-device sleep annotation algorithm.

Social interaction features, reflecting social engagement and communication patterns, were extracted from iPhone phone call and message metadata, including call and message contact counts, the number of incoming and outgoing calls and messages, and total call duration. Excluded any audio content, message content, and information about people talked to or messaged.

Speech features were derived from iPhone and Apple Watch microphone data through on-device processing, including measures of vocal prosody (pitch, jitter, shimmer, voicing, and speaking rate) as well as estimates of vocal emotion (valence, arousal, dominance, and mood) and specific vocal events (laughter, shouting). Excluded raw audio data or content of conversations.

Data from the iPhone’s front-facing camera were used, with on-device processing, to derive measurements of facial expressions (such as eyebrow raise) after device unlock and during usage of messaging apps. Excluded images or video.

Keyboard features, capturing typing dynamics from iPhone keyboard interactions, encompassed keyboard usage (frequency and duration), total emojis typed, total words typed, typing error offset, typing flight time, typing hold time, typing inter-key delay, and words altered. Sentiment analysis of typed text on the iPhone, also performed on-device, yielded features such as the frequency of absolutist words, anger emojis, anger words, anxiety emojis, anxiety words, confused emojis, and death-related words. Excluded typed content, such as specific words.

Apple Watch PPG and ECG data provided vital signs, including heart rate, heart rate variability (HRV), respiratory rate, and blood oxygenation (SpO2).

Physical activity features, such as calories burned, exercise time, stand time, and walking speed, were obtained from sensors on both devices.

Finally, device usage patterns, including usage by app categories (duration, frequency), overall screen time, and the number of unlocks, were obtained from screen interaction data from both the iPhone and Apple Watch. Excluded specific names of websites and apps, and content of notifications. Any user-initiated “mindful sessions” were also recorded as mindful minutes.

Participant privacy and data security were critically important during the design of this protocol. All data collection and processing procedures strictly adhered to relevant regulations including IRB oversight. In addition to providing informed consent, participants reviewed and authorized each data type on-device before any data were collected from their phone. Key safeguards included on-device processing to minimize the transmission of raw data. Data were deidentified before storage and analysis, utilizing unique identifiers separated from personally identifiable information. Industry-standard encryption protocols were employed for data both in transit and at rest. Secure servers with restricted access were used for data storage, following established best practices for data security.

### Medical history and biological markers

Current medication use and history of medical conditions were assessed at intake and exit. Pregnancy and childbirth history were assessed for female participants. For UCLA Health System patients, electronic health records were accessed for health conditions, hospitalizations, medications, and treatment history for five years prior to and during the study.

### Functional academic measurements

For participants who were current UCLA students, we obtained academic performance data including entrance exam scores, area of study, grades, and course enrollment.

### Data monitoring

Survey data collected through the Apple Research app were continuously exported from the app and integrated into the study REDCap to allow comprehensive data monitoring by the study team. The degree to which participants adhered to the study protocol for wearing the Apple Watch was measured using HealthKit data that participants authorized to be shared with the study. An hour was treated as compliant for watch wear if data existed in the StandHour field (either 0 or 1) for that hour. These data were uploaded to Apple, processed, and then shared with the UCLA study team. Data completeness was rigorously monitored throughout the study by members of the study team. Participants were informed at intake that the study team would contact them via email or phone regarding missing data to troubleshoot or provide reminders. Missing data criteria varied by type (e.g., three consecutive missing Daily Reflection surveys triggered a flag) (Supplementary Table 7*)*.

### Participant compensation

Participants were compensated on a tiered system at the end of each quarter based on data completion (Base: 50–55%; Silver: 67–75%; Gold: 83–89%). Tier thresholds were evaluated separately for each data stream on a weekly basis and averaged across the quarter: daily survey completion (Base: ≥4/7 days, Silver: ≥5/7, Gold: ≥6/7), watch wear ≥20 hours, and weekly overall task completion (Base: ≥58%, Silver: ≥75%, Gold: ≥85%). Watch wear was measured using HealthKit StandHour data. Full compliance requirements are detailed in Supplementary Table 7. As an example, the Gold compensation tier paid $140 per quarter for the core tasks of completing daily surveys and watch wear for 6/7 days each week, plus 85% of weekly tasks on iPhone or email each week. Longevity bonuses were introduced after six months in the study to keep participants engaged (Supplementary Fig. 8). In total, participants could earn up to $1070 for completing all components of the study over the course of one year. This total included up to $240 for completing assessment visits, up to $560 for completing the majority of frequent and recurring remote assessments (e.g., Daily Reflection Survey and watch wear), up to $160 for completing the majority of infrequent remote assessments (including the High Frequency Current Mood EMA “bursts” and Stress Logs made on the Apple Watch), and up to $110 in bonuses at the mid-point and end of the study. Additional compensation was provided for optional assessments, including hair samples, blood samples, and the diagnostic interview. Participants could keep the Apple Watch upon meeting a minimum threshold for provision of data and adherence to visits, over the entire study.

### Statistical analysis of protocol adherence and self-reported outcomes

Chi-squared tests of independence were used to examine the association between participant categories (i.e., sex at birth, age, race and ethnicity, depression severity at intake, change in depressive severity) and study completion status. One-way analysis of variance (ANOVA) was used to assess the effect of depressive status at intake on mean watch wear rates. Spearman correlations were used to estimate the associations between different self-reported outcomes.

### Pilot studies for feasibility assessment and protocol refinement

To assess the feasibility of plans for the DMHS we first performed two short pilot studies (Digital Mental Health Study Pilot 1 [DMHS-P1] and Digital Mental Health Study Pilot 2 [DMHS-P2]). We then applied lessons learned from these pilot studies in devising the DMHS protocol. The pilot studies had three main aims: (1) assess the feasibility of recruiting and retaining participants with different degrees of depression severity for longitudinal studies that passively collect digital sensing data from smartphones and smartwatches; (2) assess the feasibility of using recruitment protocols to achieve a study sample with pre-defined levels of diversity by age, sex at birth, and self-reported race and ethnicity; (3) evaluate the burden on participants of varying strategies for collecting extensive information from surveys and active tasks relating to symptoms of depression, anxiety, and stress symptoms. DMHS-P1 targeted the recruitment of 140 individuals for an eight-week study, while DMHS-P2 targeted the recruitment of 120 individuals for a 12-week study. Both pilot studies recruited all their participants from among individuals registered as patients in UCLA Health and aimed to include participants evenly distributed by age (50% over 45), sex (50% female), self-reported ethnicity (25% each Asian, Black, Hispanic, and Non-Hispanic White or Other), and current depression severity, as measured with the self-reported Computerized Adaptive Testing for Mental Health (CAT-MH)^59^ Depression severity score (50% experiencing none-to-mild depression symptoms and 50% experiencing moderate-to-severe depression symptoms).

In DMHS-P1, we used the CAT-MH to screen 1744 prospective participants online between August and November 2020, most of whom either did not complete this screening or did not fulfill eligibility requirements (Supplementary Fig. 9). Of those who were eligible and chose to enroll in the study (*N* = 184), 164 participants completed at least the first assessment session, and 159 completed the final assessment session (Supplementary Fig. 9). Eligibility criteria for DMHS-P1 are defined in Supplementary Table 8, the assessment schedule for DMHS-P1 is defined in Supplementary Fig. 10, and study procedures are defined below.

Participants were randomly assigned to one of two study conditions with respect to Ecological Momentary Assessment (EMA) questions; one condition (which included 51% of the final sample) required completion of 10 daily questions while the other condition (49% of the final sample) required completion of only four daily questions (Supplementary Table 9). All other aspects of the protocol were uniform across the sample (Supplementary Fig. 10); however, participants had the option to provide hair and saliva samples for potential biomarker analyses. The total time commitment for participants in DMHS-P1 ranged between 5.9 hours and 6.9 hours over the course of the two-month study period, reflecting the above two variables.

For this study, participants were asked to provide data for the 8-week study period, after the initial assessment session, using the provided Apple Watch and Beddit sleep tracker, and by using the Apple Research app installed on their personal iPhone or via online surveys sent throughout the study.

Overall retention rate was 96%, with 86% compliance rate for daily survey and watch wear, jointly on the same day. There was no significant difference between cohorts with and without depression symptoms, and no significant difference between the low and high burden study conditions. 87% were satisfied with the overall study experience.

The DMHS-P1 demonstrated to us that our overall approach was an efficient way to recruit and assess participants from diverse sociodemographic groups and across a wide range of depression severities. The high retention and compliance rates gave confidence that depressed patients could complete the protocol. Additionally, we determined that adoption of a relatively low burden EMA protocol could be important in achieving a high level of participant retention for a yearlong project (as envisioned for the DMHS main study).

For DMHS-P2, we screened 782 prospective participants online between March and May 2021, of whom 166 were eligible and enrolled in the study, 130 completed at least the first assessment session, and 121 completed the final assessment session (Supplementary Fig. 11).

Eligibility criteria for DMHS-P2 are defined in Supplementary Table 10, the assessment schedule for DMHS-P2 is defined in Supplementary Fig. 12, and study procedures are described below. In DMHS-P2, participants were asked to complete mood ratings 4 times per day on the watch (Supplementary Table 11), in addition to regular tasks (as often as every two weeks) administered through the Research app installed on their phone (Supplementary Fig. 12). Notable differences between the DMHS-P1 and DMHS-P2 protocols included the addition of audiovisual journals (see *Methods: Study Assessments*) and a structured diagnostic interview (NetSCID) incorporated in the intake assessment session for all participants in the latter study. The addition of the diagnostic interview lengthened this intake session substantially, from approximately 3 hours in DMHS-P1 to approximately 5.5 hours in DMHS-P2. Furthermore, DMHS-P2 participants were issued two watches and asked to wear a watch continuously throughout the study duration (wearing one watch while the other was charged, including overnight). The time commitment for participants enrolled in DMHS-P2 was approximately 12 hours over the study period (12–14 weeks).

Participants were asked to provide data for the 12-week study period, after the initial assessment session, using the provided study equipment, which included two Apple Watches and the Beddit sleep tracker. Participants were asked to always wear one of the two Apple Watches for continuous day and night data collection and to use the Beddit sleep tracker for the 12-week study period. Participants were again asked to complete tasks using the Apple Research app installed on their personal iPhone or via online surveys sent throughout the study. In addition to the longer study duration, new assessments were added to the study protocol following DMHS-P1. Participants completed weekly Video Journals via the Apple Research app installed on their phone; participants had up to five minutes to record themselves responding to the following three prompts: “1 How are you feeling today? 2 Did you feel angry, stressed, or sad this week? If so, why? 3 Did something good happen this week? If so, what was it?”. Participants were asked to provide ad hoc mood timestamps using the Apple Watch. In addition to daily symptom assessments (where all participants now received 4 questions daily, Supplementary Table 11), participants were asked to complete a weekly symptom survey via the Apple Research app. In addition to the clinical rating scales to assess depression and anxiety, all participants also completed a structured diagnostic interview (NetSCID) as part of their intake assessment session at the beginning of the study. In this phase, half of the sample was selected to be offered the option of providing hair or saliva samples near the intake and exit assessment sessions for the measurement of cortisol for additional compensation. Sample collection kits were shipped to participants and returned to the research team by mail.

Based on DMHS-P2 we determined that an interviewer-based diagnostic assessment (i.e., NetSCID) was not scalable for the full DMHS and therefore decided to use a self-report DSM-5 diagnostic assessment (SAGE-SR) and reserve the NetSCID for a selected sub-sample (as described in *Methods*). Additionally, we decided that a watch-wearing protocol based on the use of two Apple Watches was too complex, and therefore instituted the simplified protocol described in *Methods*. Participants reported that they preferred to have all their tasks on one device, with one place to track completion. The EMA task was therefore reverted to the iPhone for the main DMHS study, with an optional watch survey task for bonus compensation (Stress Logs). We also learned that the Video Journal prompts often elicited very short responses (with means 15, 27, and 24 seconds). Questions 1 and 3 were therefore updated to the wording shown in Supplementary Table 5B to elicit longer responses in the main DMHS study.

## Supplementary information

Douglas_npjDigitalPublicHealth_Supplement010826

## Data Availability

Data are not publicly available. Limited data access to support the findings of this paper may be available to research institutions. Any request for data access may be evaluated and responded to in a manner consistent with policies intended to protect participant confidentiality, language in the study protocol and informed consent form, and other applicable agreements with Apple. Requests for data access should be addressed to the corresponding author (N.B.F.).

## Code availability

Code used to generate the summary statistics and figures reported in this article is not publicly posted because it is linked to restricted study data structures. Annotated code sufficient to reproduce the reported analyses on approved-access data is available from the corresponding author upon reasonable request and subject to applicable approvals and agreements.
